# Exploring the risk factors of early sepsis after liver transplantation: development of a novel predictive model

**DOI:** 10.3389/fmed.2023.1274961

**Published:** 2023-11-29

**Authors:** Wanting Chen, Shengdong Wu, Lingwen Gong, Yu Guo, Li Wei, Haoran Jin, Yan Zhou, Chuanshuang Li, Caide Lu, Lanman Xu

**Affiliations:** ^1^Department of Infectious Diseases and Liver Diseases, Ningbo Medical Centre Lihuili Hospital, Affiliated Lihuili Hospital of Ningbo University, Ningbo, China; ^2^Department of Hepatopancreatobiliary Surgery, Ningbo Medical Center Lihuili Hospital, Affiliated Lihuili Hospital of Ningbo University, Ningbo, China; ^3^Department of Infectious Diseases, Zhejiang Provincial Key Laboratory for Accurate Diagnosis and Treatment of Chronic Liver Diseases, The First Affiliated Hospital of Wenzhou Medical University, Hepatology Institute of Wenzhou Medical University, Wenzhou, China

**Keywords:** sepsis, liver, transplantation, risk factors, predictive model

## Abstract

**Background:**

Sepsis is a severe and common complication of liver transplantation (LT) with a high risk of mortality. However, effective tools for evaluating its risk factors are lacking. Therefore, this study identified the risk factors of early post-liver transplantation sepsis and established a nomogram.

**Methods:**

We analyzed the risk factors of post-liver transplantation sepsis in 195 patients. Patients with infection and a systemic inflammatory response syndrome (SIRS) score ≥ 2 were diagnosed with sepsis. The predictive indicators were screened with the least absolute shrinkage and selection operator (LASSO) and collinearity analyses to develop a nomogram. The prediction performance of the new nomogram model, Sequential Organ Failure Assessment (SOFA) score, and Modified Early Warning Score (MEWS) was compared through assessment of the area under the curve (AUC), decision curve analysis (DCA), net reclassification index (NRI), and integrated discrimination improvement (IDI).

**Results:**

The nomogram was based on postoperative heart rate, creatinine concentration, PaO_2_/FiO_2_ ratio < 400 mmHg, blood glucose concentration, and international normalized ratio. The AUC of the nomogram, the SOFA score, and MEWS were 0.782 (95% confidence interval CI: 0.716–0.847), 0.649 (95% CI: 0.571–0.727), and 0.541 (95% CI: 0.469–0.614), respectively. The DCA curves showed that the net benefit rate of the nomogram was higher than that of the SOFA score and MEWS. The NRI and IDI tests revealed better predictive performance for the nomogram than SOFA score and MEWS.

**Conclusion:**

Heart rate, creatinine concentration, PaO_2_/FiO_2_, glucose concentration, and international normalized ratio should be monitored postoperatively for patients at risk of post-liver transplantation sepsis. The nomogram based on the aforementioned risk factors had a better predictive performance than SOFA score and MEWS.

## 1 Introduction

Liver disease leads to approximately 2 million deaths each year globally (1), imposing a financial burden on the healthcare system. Conventional medical treatments can improve the prognosis in some patients. Of these, liver transplantation (LT) is the most effective life-saving treatment for end-stage liver diseases (2); however, the management of postoperative complications remains challenging. Infections and sepsis in particular are the primary causes of postoperative mortality during the first month following LT (3).

Sepsis is a life-threatening organ dysfunction caused by the dysregulated host response to infection (4). Patients who develop infections or sepsis after LT may experience deterioration in their health. This may prolong hospitalization and increase financial burden, which may result in severe outcomes and even death. A retrospective analysis revealed that the incidence of sepsis after LT is as high as 50%–80%, and that sepsis-related deaths account for 50–90% of all postoperative mortality (5–9). According to the Surviving Sepsis Campaign recommendations, early fluid resuscitation and timely initiation of antibiotics are cornerstones for the successful treatment of patients with sepsis (10). Therefore, early and accurate prediction of sepsis can help clinicians to intervene promptly and significantly improve the prognosis of LT. Studies have reported that exhaled nitric oxide, aerobic capacity (11), plasma amino acid profile (12), and muscle wasting (13) are indicators for sepsis after LT. However, these indicators cannot be easily identified in clinical practice. Therefore, simple, precise, and specific predictive models for early detection of post-LT sepsis are needed.

This study aimed to analyze the risk factors for early sepsis and develop a nomogram for early sepsis after LT.

## 2 Materials and methods

### 2.1 Patients

In this study, we retrospectively enrolled 227 consecutive patients who underwent LT at the Ningbo Medical Center, LiHuiLi Hospital (Zhejiang, China), between January 2016 and December 2021. The study was approved by the hospital ethics committee (KY2022PJ026).

**Figure 1:**
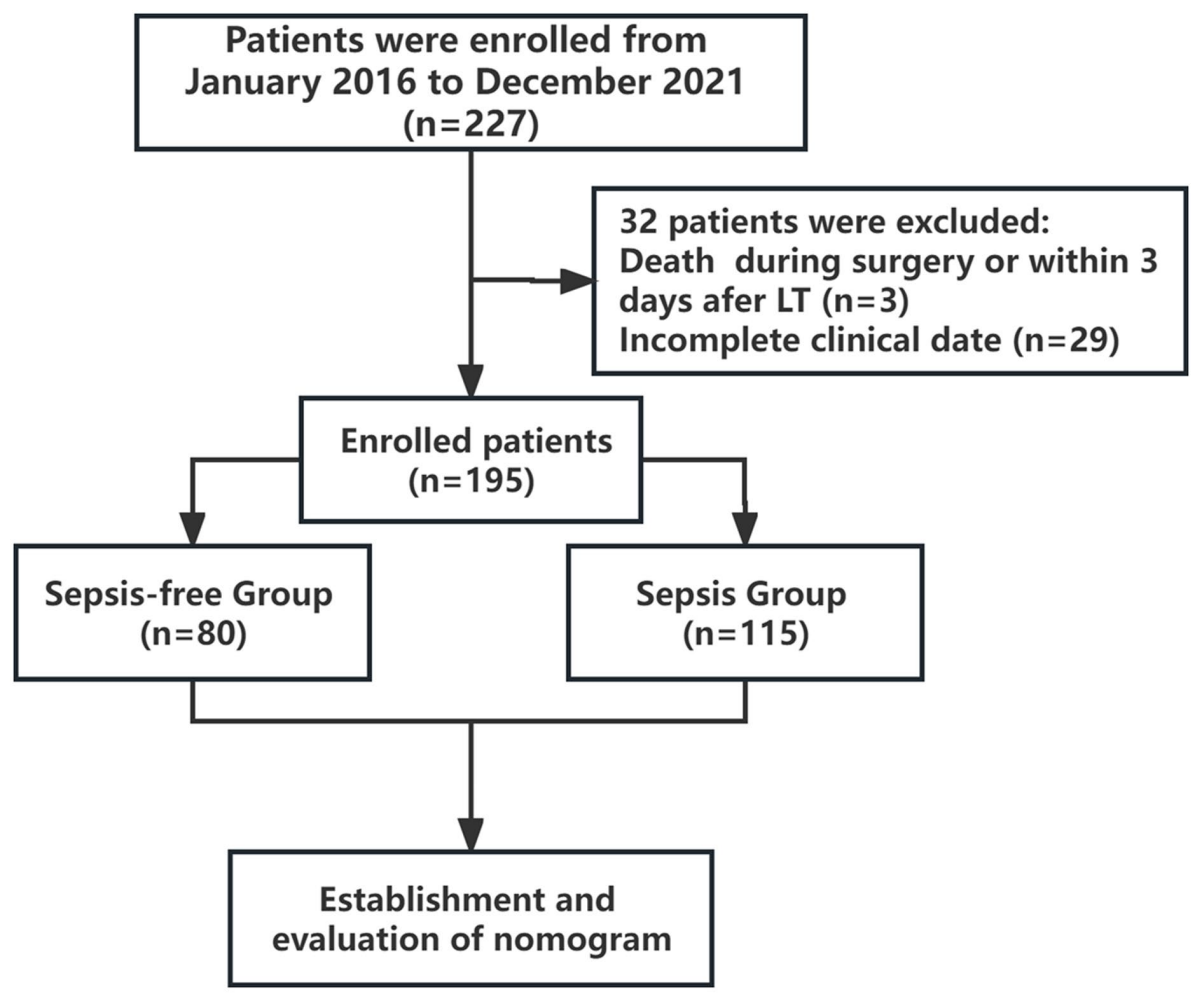
Study flow chart. The enrolled patients were divided into sepsis and sepsis-free groups within two weeks after LT.

Patients were excluded if they met the following criteria: age of <18 years or > 65 years, death during surgery or within 3 days after LT (*n* = 3), or incomplete clinical data (*n* = 29). Finally, 195 patients were enrolled and classified into the sepsis and sepsis-free groups (Figure 1). Patients with infection and a systemic inflammatory response syndrome (SIRS) score ≥ 2 were diagnosed with sepsis. Infection is defined as a microbial phenomenon characterized by an inflammatory response to the presence of microorganisms or the invasion of normally sterile host tissue by those organisms. The diagnostic criteria for SIRS are two or more of the following clinical manifestations: heart rate > 90 bpm; body temperature<36°C or > 38°C; white blood cell count <4 × 10^9^/L or > 12 × 10^9^ /L; respiratory rate > 20 cycles/min or arterial partial pressure of carbon dioxide (PaCO_2_) <32 mmHg (1 mmHg = 0.133 kPa) (14).

### 2.2 Prevention and treatment using antibiotics

During the perioperative period, β-lactam antibiotics were used to prevent postoperative infection in most patients.

### 2.3 Immunosuppressive regimen

Recipients were administered baliximab 30 min before LT and on day 4 postoperatively. Methylprednisolone (500 mg) was administered once at the beginning of the hepatic-free period. The postoperative immunosuppressive regimen comprised tacrolimus in combination with mertilmicosporin.

### 2.4 Artificial liver support system mode

The artificial liver was used to treat patients with end-stage liver disease before LT. The main modes included plasma replacement or a combination of plasma replacement and continuous renal replacement therapy (CRRT). In the sepsis group, plasma replacement alone was employed for eight cases and plasma replacement combined with CRRT for ten. In contrast, in the sepsis-free group, three cases utilized plasma replacement alone, whereas two utilized plasma replacement in conjunction with CRRT.

### 2.5 Clinical data collection and risk factors

The general clinical data included age, sex, primary disease, surgical method, invasive operations (including surgery, interventional therapy, artificial liver, kidney replacement therapy, and various forms of paracentesis), and artificial liver treatment within 1 month before LT. In our study, the primary diseases that necessitated LT included hepatocellular carcinoma (including hepatocellular carcinoma complicated with liver cirrhosis), liver cirrhosis, and acute liver failure, among others.

The preoperative laboratory indicators were as follows: heart rate (HR), mean arterial pressure (MAP), white blood cell (WBC) count, Neutrophil to Lymphocyte ratio (NLR), hemoglobin concentration (Hb), platelet count (PLT), albumin concentration (ALB), total bilirubin (TBil), creatinine concentration (Cr), prothrombin time (PT), international normalized ratio (INR), serum sodium concentration (Na^+^), and model for end-stage liver disease (MELD). The intraoperative indicators were as follows: anhepatic phase, blood loss, and plasma transfusion volume. The postoperative laboratory indicators were the first values of the following measures on postoperative day (POD) 3: HR, MAP, Systolic blood pressure (SBP), shock index (SI = HR-to-SBP ratio), use of hyperensort (including norepinephrine, dopamine, and mesalamine), WBC, NLR, Hb, PLT, ALB, TBil, Cr, PT, INR, arterial partial oxygen pressure (PaO_2_), fraction of inspired O_2_ (FiO_2_), PaO_2_-to-FiO_2_ ratio (PaO_2_/FiO_2_), lactic acid concentration (Lac), C-reactive protein concentration (CRP), Blood FK506 concentration, blood glucose concentration (Glu), abnormal liver blood supply (abnormalities of hepatic arteriovenous blood flow and hepatic ischemia), and hydropericardium. Laboratory results were collected on the day before transplantation and POD 3. Notably, CRP peaked within 3 days after LT, and blood concentration of FK506 was collected from patients 7 days after LT. We also investigated the characteristics of pathogenic bacteria distribution in patients with sepsis after LT.

### 2.6 Establishment and evaluation of prediction model

We employed least absolute shrinkage and selection operator (LASSO) regression to screen the variables and generalized linear modeling to obtain regression coefficients, odds ratios (OR), and 95% confidence intervals (CI) for the modeled variables.

The area under the curve (AUC) was used to evaluate the discriminative ability of the nomogram. Hosmer–Lemeshow (H-L) test was used to assess goodness-of-fit. Calibration curves were plotted based on the predicted and true probabilities. We assessed the nomogram performance and compared it to SOFA scores and MEWS using metrics such as the area under the curve (AUC), decision curve analysis (DCA), net reclassification index (NRI) and integrated discrimination index (IDI). NRI was calculated using the nricens package. The cut points were selected as 0.3 and 0.7, and 1,000 resampling iterations were performed using the bootstrap method (niter = 1,000). The updown parameter was configured to assess categories, distinguishing between low, medium and high risk categories.

### 2.7 Statistical analysis

Statistical analyses were performed with R 4.0.3 (https://www.r-project.org/; The R Foundation). Normally distributed variables, non-normally distributed variables, and categorical variables were expressed as mean and standard deviation, median (first quartile, third quartile), and frequency (percentage), respectively. The independent *t*-test was used to compare the normally distributed variables. Mann–Whitney *U*-test was applied to the non-normally distributed variables, and the chi-squared test was used to compare the categorical variables. Univariate analysis was used to screen for the risk factors of sepsis, and the Youden index was used to determine the optimal cutoff values for the independent risk factors. *p* < 0.05 was considered statistically significant.

## 3 Results

### 3.1 Clinical characteristics of patients

We included 195 patients (157 males and 38 females) in this study. The average age was 52.29 ± 9.45 years, and the indications for LT were liver cancer (*n* = 91, 46.67%), liver cirrhosis (*n* = 96, 49.23%), liver failure (*n* = 5, 2.56%), and other diseases (*n* = 3, 1.54%). The pathogenic factors included viral hepatitis B (*n* = 147, 75.38%), alcoholic liver injury (*n* = 12, 6.15%), autoimmune liver disease (*n* = 15, 7.69%), and others (*n* = 21, 10.77%). The main surgical methods used were classical orthotopic LT (*n* = 144, 73.85%), piggyback liver allograft transplantation (*n* = 26, 13.34%), split LT (*n* = 17, 8.72%), and living donor LT (*n* = 8, 4.10%). A total of 116 recipients of LT developed sepsis within 1 month, with 115 cases occurring within the first 2 weeks after transplantation. The median time to sepsis onset was 5 days.

As shown in Supplementary Table S1, there were no significant differences between the sepsis and sepsis-free groups (*p* > 0.05) in gender, age, preoperative infection, prevalence of diabetes mellitus, invasive operations, preoperative indicators [WBC (*z* = −0.676, *p* = 0.499), NLR (*z* = −0.132, *p* = 0.895), Hb (*t* = 1.158, *p* = 0.254), PLT (*z* = 0.588, *p* = 0.556), ALB (*t* = −0.392, *p* = 0.699), TBil (*z* = −1.351, *p* = 0.176), Na^+^ (*t* = 1.198, *p* = 0.232), Cr (*z* = 0.464, *p* = 0.642), PT (*t* = −0.733, *p* = 0.464), INR (*z* = −0.788, *p* = 0.431), HR (*t* = 1.099, *p* = 0.273) and MAP (*t* = 0.749, *p* = 0.455)], intraoperative indicators [anhepatic phase (*z* = −1.534, *p* = 0.125), blood loss (*z* = −1.726, *p* = 0.084), and plasma transfusion volume (*z* = −1.245, *p* = 0.213)], postoperative indicators [SBP (*t* = 0.120, *p* = 0.903), use of hyperensort (*χ^2^* = 2.953, *p* = 0.086), MAP (*t* = −0.079, *p* = 0.067), WBC (*t* = −1.941, *p* = 0.067), Hb (*t* = 0.996, *p* = 0.322), PLT (*t* = 1.169, *p* = 0.231), CRP (*z* = −1.498, *p* = 0.134), NLR (*z* = −0.168, *p* = 0.536), Blood FK506 concentration (*t* = 1.299, *p* = 0.195), abnormal liver blood supply (*χ^2^* = 0.039, *p* = 0.844), hydropericardium (*χ^2^* = 2.898, *p* = 0.089), and length of intensive care unit (ICU) stay (*t* = −1.902, *p* = 0.105)]. However, in the sepsis group, the proportion of patients who received artificial liver treatment before LT (*χ^2^* = 4.00, *p* = 0.045), the proportion of patients with a MELD score > 20 before LT (*χ^2^* = 3.92, *p* = 0.048), and the proportion of patients with PaO_2_/FiO_2_ < 400 mmHg (*χ^2^* = 14.23, *p* < 0.001) after LT were higher than those in the sepsis-free group. These differences between groups were statistically significant. Meanwhile, HR (*t* = −3.56, *p* = 0.001), SI (*t* = −2.79, *p* = 0.007), TBil (*z* = −2.71, *p* = 0.007), Cr (*z* = −2.99, *p* = 0.003), INR (*z* = −4.26, *p <* 0.001), Lac (*z* = −3.74, *p* < 0.001), and Glu (*z* = −2.35, *p* = 0.012) were significantly higher for the sepsis group on POD 3 than that for the sepsis-free group (*p* < 0.05). Conversely, the postoperative ALBs for the sepsis group were significantly lower than those for the sepsis-free group (*t* = −0.39, *p* = 0.046).

AUC was used to evaluate the efficiency of the indicators in discriminating between sepsis and sepsis-free groups. The Youden index, which is defined as the maximum vertical distance between the receiver operating characteristic (ROC) curve and the diagonal line, was used to determine the optimal cutoff value. As shown in Table 1, the optimal cutoff values for the risk factors of sepsis were ALB <33.7 g/L, HR ≥ 90 bpm, HR/SBP ≥ 0.64, TBil ≥79.25 μmol/L, Cr ≥ 89 μmol/L, INR ≥ 1.52, Lac ≥2.75 mmol/L, Glu ≥ 16.50 mmol/L, and PaO_2_/FiO_2_ < 400 mmHg on the third day after LT (*p* < 0.05).

**Table 1:** Statistical differences in indicators between the sepsis-free and sepsis groups after liver transplantation.

Variable		Sepsis-free group (*n* = 80)	Sepsis group (*n* = 115)	χ^2^	*p-value*
ALB (g/L)	<33.7	14 (17.5%)	74 (64.3%)	7.677	0.006
HR (bpm)	≥90	30 (37.5%)	75 (65.2%)	14.585	<0.001
HR/SBP	≥0.64	47 (58.8%)	89 (77.4%)	7.769	0.005
TBil (μmol/L)	≥79.25	28 (35.0%)	67 (58.3%)	10.218	0.001
Cr (μmol/L)	≥89.00	23 (28.8%)	62 (53.9%)	12.149	<0.001
INR	≥1.52	13 (22.4%)	81 (70.4%)	15.247	<0.001
Lac (mmol/L)	≥2.75	33 (41.3%)	76 (66.1%)	11.806	0.001
Glu (mmol/L)	≥16.5	25 (18.8%)	46 (40.0%)	9.911	0.002
PaO_2_/FiO_2_ (mmHg)	<400	25 (43.1%)	102 (88.7%)	9.037	0.003

ALB, albumin; HR, heart rate; HR/SBP, heart rate to systolic blood pressure ratio; TBil, total bilirubin; Cr, creatinine; INR, international normalized ratio; Lac, lactic acid; Glu, blood glucose; PaO_2_/FiO_2,_ PaO_2_-to-FiO_2_ ratio.

### 3.2 Prevention and treatment using antibiotics

During the LT perioperative period, β-lactam antibiotics (*n* = 75, 93.80%), enzyme inhibitors (*n* = 2, 2.50%), β-lactam antibiotics combined with antifungals (*n* = 2, 2.50%), and β-lactam antibiotics combined with glycopeptide antibiotics (*n* = 1, 1.30%) were used as prophylactic treatment for the sepsis-free group. Furthermore, β-lactam antibiotics (*n* = 103, 89.60%), enzyme inhibitors (*n* = 1, 0.90%), β-lactam antibiotics in combination with antifungals (*n* = 10, 8.70%), and β-lactam antibiotics in combination with glycopeptide antibiotics (*n* = 1, 0.90%) were used as prophylactic treatment for the sepsis group. These differences between two groups were not statistically significant (*χ2* = 3.20, *p* = 0.271).

### 3.3 Characteristics of the distribution of pathogenic bacteria in early sepsis following LT

In the sepsis group, 19 (16.52%) of the patients had mixed infections with two or more bacteria, 13 (11.30%) had infections with multiple flora at a single site, and 33 (28.70%) had infections at multiple sites. The most common being gram-negative strains (*n* = 26, 22.60%) dominated by *Acinetobacter baumannii* (*n* = 8, 30.77%, including five multi/pan-drug resistant strains); gram-positive strains (*n* = 15, 13.04%), dominated by *Enterococcus faecium* (*n* = 10, 66.67%), and fungi (*n* = 17, 14.78%), dominated by *Candida albicans* (*n* = 4, 23.53%). The most common site of infection in patients with sepsis was the lungs (*n* = 70, 60.87%).

### 3.4 Development and validation of the predictive nomogram

**Figure 2:**
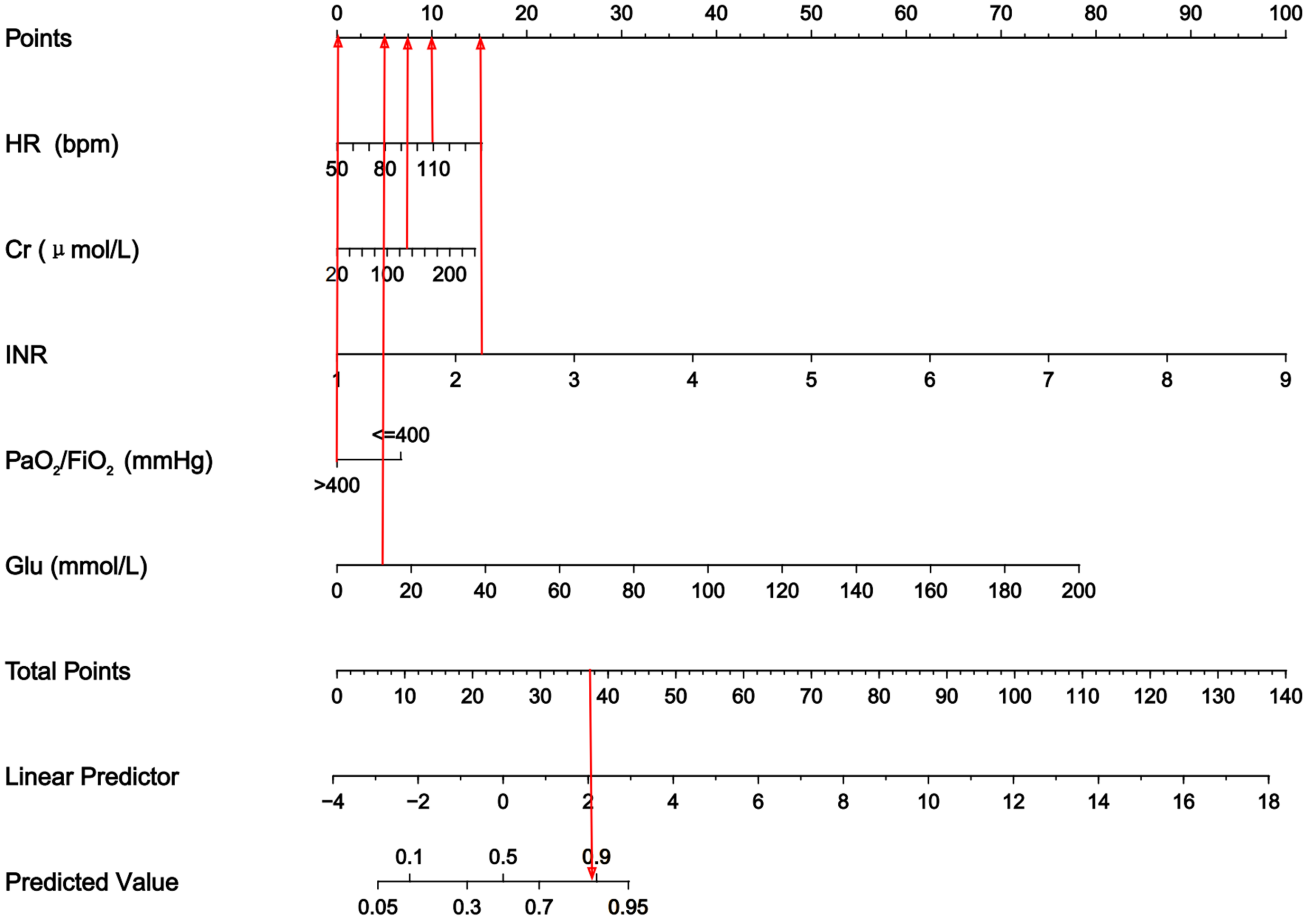
Nomogram to predict the risk of sepsis in patients within two weeks following LT. The nomogram assigned a specific score on the point scale axis for each variable, and these individual scores were summed to calculate the total score. This total score can be projected to estimate the risk of early sepsis after LT. HR, heart rate; Cr, creatinine; INR, international normalized ratio; PaO_2_/FiO_2_, PaO_2_-to-FiO_2_ ratio; Glu, blood glucose.

We used LASSO logistic regression analysis to screen for the predictors of sepsis. We identified five significant predictors (Table 2), including postoperative HR (95% CI: 1.006–1.050, *p* = 0.012), postoperative Cr (95% CI: 1.000–1.023, *p* = 0.067), postoperative INR (95% CI: 2.396–25.568, *p* = 0.001), postoperative PaO_2_/FiO_2_ < 400 mmHg (95% CI: 1.527–5.681, *p* = 0.001), and postoperative Glu (95% CI: 1.000–1.147, *p* = 0.089), which were used to establish the prediction nomogram for sepsis within 2 weeks after LT (Figure 2).

**Table 2:** LASSO logistic regression analysis of the study population.

Variable	*Β*	OR	95% CI	*p-value*
HR	0.027	1.028	1.006–1.050	0.012
Cr	0.011	1.011	1.000–1.023	0.067
INR	1.992	7.329	2.396–25.568	0.001
PaO_2_/FiO_2_ < 400	1.071	2.917	1.527–5.681	0.001
Glu	0.062	1.064	1.000–1.147	0.089

The variables presented in the table were collected on postoperative day 3 after liver transplantation. HR, heart rate; Cr, creatinine; INR, international normalized ratio; PaO_2_/FiO_2_, PaO_2_-to-FiO_2_ ratio; Glu, blood glucose; OR, odds ratio; CI, confidence interval; LASSO, least absolute shrinkage and selection operator.

### 3.5 Evaluation of established prediction model

#### 3.5.1 Discriminative ability and validity of the nomogram

**Figure 3:**
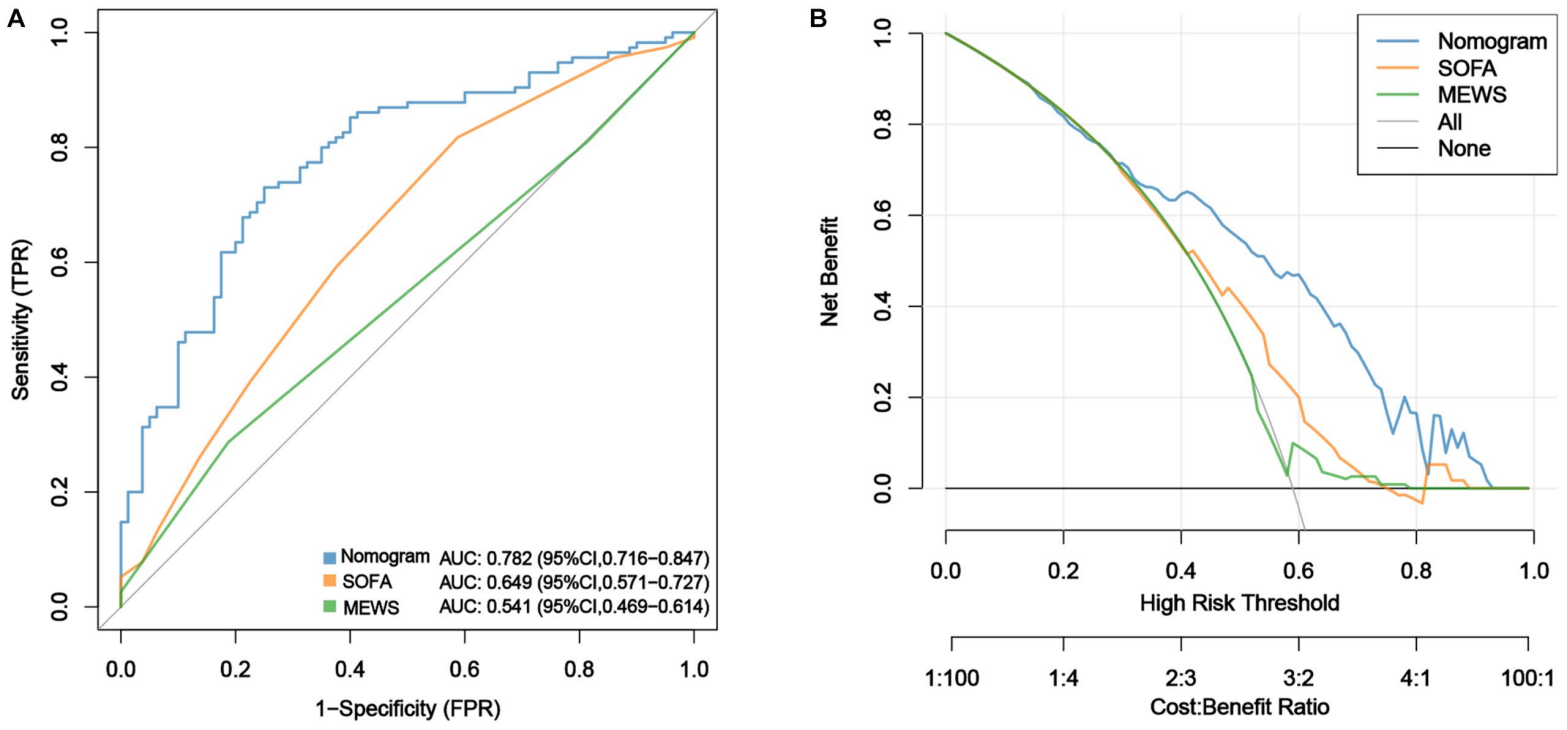
Comparison of the performances of the nomogram, SOFA score and MEWS in predicting sepsis. **(A)** ROC curves of the nomogram, SOFA score and MEWS in predicting risk of sepsis within two weeks after LT and **(B)** DCA for evaluation of the validity ability of the nomogram, SOFA score and MEWS.

**Figure 4:**
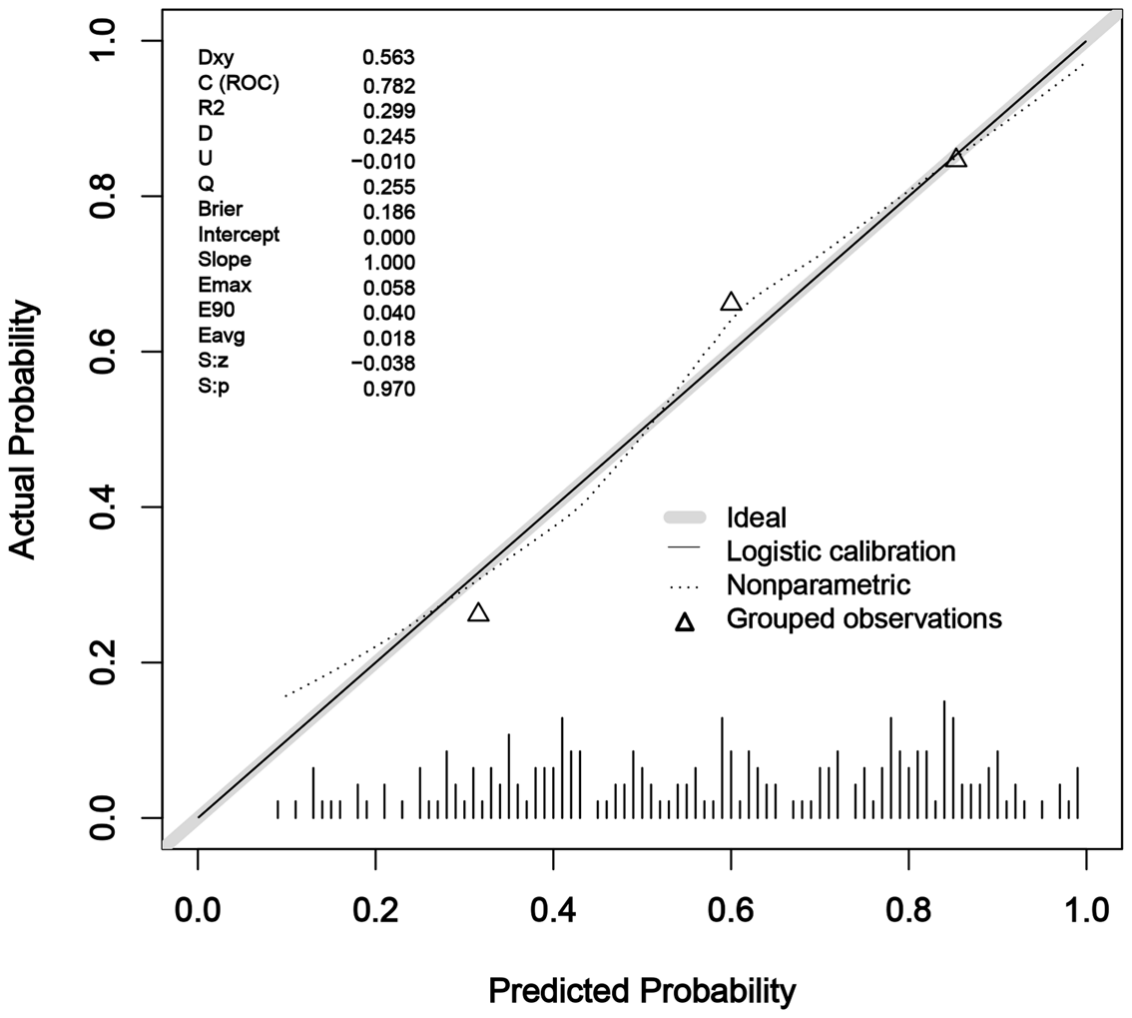
Calibration plot of nomogram in predicting sepsis after LT.

ROC analysis was used to evaluate the discriminatory power of the nomogram, and the AUC of the nomogram was 0.782 (95% CI, 0.716–0.847) (Figure 3A). The H-L test showed a good fit of the actual data (χ2 = 9.793, *p* < 0.05). Furthermore, the calibration plots demonstrated a high level of concordance between the predicted and actual probabilities (Figure 4).

Decision curve analysis (DCA) was used to assess the validity and clinical usefulness of the nomogram. The decision curve of the nomogram was situated above the two extreme curves (Figure 3B), indicating an overall net benefit within a broad threshold probability range of 30%–80%.

#### 3.5.2 Comparison of the performances of the nomogram SOFA score and MEWS in predicting sepsis

The AUCs of the SOFA scores and MEWS were 0.649 (95% CI, 0.571–0.727) and 0.541 (95% CI, 0. 469–0.614), respectively, indicating that the nomogram demonstrated superior predictive performance for early sepsis (Figure 3A). The DCA curves showed that the net benefit rate of the nomogram was higher than that of the SOFA score and MEWS with the threshold in the range of 0.30–0.95 (Figure 3B). Further, the nomogram had a markedly higher discriminative power than the SOFA score and MEWS, with NRI values of 1.973 (95% CI: 1.668–1.823, *p* < 0.001) and 1.973 (95% CI: 1.941–2.000, *p* < 0.001), and IDI values of 0.171 (95% CI: 0.118–0.225, *p* < 0.001) and 0.222 (95% CI: 0.160–0.284, *p* < 0.001), respectively. Taken together, the nomogram developed in this study may be effective for predicting early sepsis in clinical practice.

## 4 Discussion

As LT is one of the most effective treatments for end-stage liver diseases, 34,694 LTs were performed globally in 2021, according to the International Registry on Organ Donation and Transplantation. However, the complications of LT, especially sepsis, are associated with a high risk of mortality (15, 16). A single-center retrospective study showed that the incidence of early sepsis after LT was up to 67%, and the median time to infection was 9 days (6, 17). The present study enrolled 195 patients who underwent LT, and found the median time to sepsis to be 5 days and the prevalence of early sepsis after LT to be 59%, which is much higher than the incidence of sepsis in other diseases in the intensive care unit (1, 18). The high incidence of sepsis may be related to intraoperative ischemia–reperfusion injury (19, 20), length of ICU stay (21), invasive procedures (22, 23), and high postoperative doses of hormones and immunosuppressants (24), among others. Early source control in sepsis (<6 h) is associated with a reduced risk-adjusted odds of 90-day mortality (25–27). Therefore, clinical data on POD 3 were collected in the present study due to the shorter incubation period (28). Bacterial infections are prevalent at first 30 days after LT (29, 30), which account for up to 70% of all infections in liver transplant recipients (31). In this study, *Enterococcus faecalis* was the most common pathogen isolated in the sepsis group after LT (17.24%). *Enterococcus faecium* bloodstream infections (BSI) is an independent risk factor for death compared to other bacterial BSI. And the mean 30-day mortality rate for *Enterococcus faecium* BSI is high to 25% (32). However, differentiating sepsis from non-infectious conditions is difficult, especially in patients who may develop systemic inflammatory response syndrome after LT. Therefore, establishing an effective predictive model for sepsis after LT is critical.

A previous study reported that the SOFA score and CRP can be combined to evaluate the risk of sepsis after LT (33). However, this is controversial because of the use of sedative and analgesic drugs after LT, which could affect the Glasgow Coma Scale score included in the SOFA score. Meanwhile, the most commonly used markers of inflammation such as C-reactive protein and procalcitonin, are unable to represent the development of sepsis because of factors affecting preoperative recipient status and the intraoperative and postoperative courses, particularly during the early stages after LT (34, 35). The MEWS is a commonly used scoring system for sepsis severity (36). Nonetheless, its applicability in predicting sepsis following LT may be limited due to its inclusion of a consciousness score. As mentioned above, there was no significant difference in the CRP between the sepsis and sepsis-free groups in our study, which may be attributed to the severe trauma caused by LT, high doses of antibiotics, and immunosuppressants that prevent inflammatory indicators from reflecting the infection status of the patient.

Nomograms have emerged as valuable tools in various fields, enabling clinicians to predict the likelihood of clinical events based on individual variables. In this study, we identified postoperative HR, Cr, PaO_2_/FiO_2_ < 400 mmHg, Glu, and INR as independent risk factors for sepsis after LT. The AUC for the nomogram was 0.782, demonstrating excellent performance in distinguishing between patients who will or will not develop sepsis within 2 weeks after LT.

Tachycardia is one of the most important indicators in the diagnostic criteria of sepsis (14) and is widely acknowledged as a robust indicator of sepsis-related morbidity and mortality (37–39). By analyzing the ROC curves, we determined that an HR threshold of 90 bpm was ideal for predicting the risk of sepsis, which is consistent with previous reports of research on multiorgan dysfunction syndrome (40, 41). Tachycardia is an early and reliable sign of hypotension that helps in sustaining sufficient cardiac output and circulating volume (10). However, in our study, the increase in heart rate was not accompanied by hypotension. Hypotension is a condition that may be associated with inflammation, which leads to vasodilatation, a decrease in preload, and an overall decrease in cardiac output (42). This results in the activation of sympathetic nerves, leading to compensatory tachycardia and vasoconstriction.

A previous study showed the PaO_2_/FiO_2_ ratio is crucial for assessing the risk of mortality and severity of sepsis (43, 44). Our study also showed that PaO_2_/FiO_2_ ratios below 400 mmHg were significant risk indicators for sepsis, and 88.7% of patients in the sepsis group had PaO_2_/FiO_2_ ratios below 400 mmHg. Previously, preoperative and intraoperative inflammatory response syndrome, perioperative massive blood transfusion, and ischemia–reperfusion injury have been identified as risk factors for postoperative hypoxemia (45). Rational interventions and timely treatment of hypoxemia are essential to reduce the morbidity of sepsis.

Based on the laboratory test results, Glu has emerged as a potential risk factor for sepsis (46, 47). One study reported that the peak Glu can be used as an adjunctive tool for mortality risk stratification in critically ill patients with sepsis (48, 49). Patients with end-stage liver disease have impaired glucose metabolism (50). In most cases, Glu rapidly decreases within 2 days after LT (51). Our study found that the risk of sepsis was significantly increased when Glu was ≥16.5 mmol/L on POD 3. Controlling hyperglycemia can be a challenging task, however employing a number of measures preoperatively can help control a patient’s intraoperative and postoperative glucose levels and reduce postoperative infections (52).

INR is also an effective sepsis screening indicator and prognostic factor (53, 54). During the initial stages of infection, coagulation acts as a natural defense mechanism in an attempt to limit the pathogen and prevent its spread to systemic circulation (48, 55). Studies have shown that coagulation function is abnormal in severe infections, and the main reason for its formation is the imbalance between the formation and clearance of fibrin in the blood vessels (56). Patients with INR exceeding 1.52 on POD 3 had considerably heightened susceptibility to sepsis—70.4% of the patients in this study had INR exceeding 1.52. In addition, renal dysfunction is a prevalent complication of LT. A recent study by Mehta et al. showed that 40% of critically ill patients developed sepsis after acute kidney injury (57). In this study, patients with sepsis had a high Cr, with a value of 93.2 μmol/L on POD 3. In contrast, the patients in the sepsis-free group had a lower Cr (73.4 μmol/L) than the sepsis group (93.2 μmol/L). Furthermore, a significant association was established between Cr exceeding 89 μmol/L and the likelihood of sepsis.

To further validate the predictive performance of the sepsis nomogram developed in this study, we compared its performance to that of the SOFA score and MEWS. The nomogram demonstrated a higher prediction accuracy and net benefit than the SOFA score and MEWS. These suggest that our nomogram is practical and viable for predicting postoperative sepsis in patients undergoing LT. The high prognostic value of the nomogram is likely attributable to its ability to combine the prognostic values of renal, respiratory, and liver insufficiency. Furthermore, this study highlighted the potential benefits of personalized medicine and the value of predictive models in improving patient outcomes. Sepsis risk assessment is no longer abstract, and clinical prevention and treatment should focus on patients with these characteristics.

This study had a few limitations. First, the data were retrospectively collected from the medical records of the patients, which may not have contained all the necessary information for analysis. Second, the generalization of the study findings may be limited due to the small sample size, and external validation is required to validate our findings.

## 5 Conclusion

In conclusion, this study identified the risk factors for sepsis within 2 weeks after LT and developed a novel nomogram for predicting sepsis. This nomogram can facilitate early identification of sepsis and enable timely clinical intervention, thereby improving the prognosis of patients undergoing LT.

## Data availability statement

The original contributions presented in the study are included in the article/Supplementary material, further inquiries can be directed to the corresponding authors.

## Ethics statement

The studies involving humans were approved by Ningbo Medical Centre Lihuili Hospital. The studies were conducted in accordance with the local legislation and institutional requirements. The ethics committee/institutional review board waived the requirement of written informed consent for participation from the participants or the participants’ legal guardians/next of kin because this is a retrospective study that primarily utilizes available clinical data to retrospectively summarize and analyze.

## Author contributions

WC: Conceptualization, Formal analysis, Supervision, Writing – original draft. SW: Methodology, Resources, Writing – original draft. LG: Data curation, Resources, Writing – original draft. YG: Investigation, Software, Writing – original draft. LW: Formal analysis, Software, Writing – original draft. HJ: Investigation, Writing – original draft. YZ: Writing – original draft, Data curation. ChL: Visualization, Writing – original draft. CDL: Writing – review & editing. LX: Conceptualization, Writing – review & editing.
