# Supplementary material for: Exploring the risk factors of early sepsis after liver transplantation: development of a novel predictive model
Source: Front Med (Lausanne). 2023 Nov 29;10:1274961. doi: 10.3389/fmed.2023.1274961 (PMC10716451; doi:10.3389/fmed.2023.1274961)
Supplement: Supplementary file 1 [file Table_1.docx]

***Supplementary Material***

**Supplementary Table 1: Clinical characteristics of patients**

| Variables | Total Population  (n = 195) | Sepsis-free Group  (n = 80) | Sepsis Group  (n = 115) | P-value |
| --- | --- | --- | --- | --- |
| Age | 52.29 ± 9.45 | 53.08 ± 8.86 | 51.75 ± 9.83 | 0.336 |
| Sex, Male | 157 (80.5%) | 65 (81.2%) | 92 (80.0%) | 0.828 |
| **Preoperative indicators** |  |  |  |  |
| Infection | 45 (23.1%) | 16 (20.0%) | 29 (25.2%) | 0.395 |
| Diabetes mellitus | 32 (16.4%) | 13 (16.2%) | 19 (16.5%) | 0.960 |
| Invasive operations | 28 (14.4%) | 9 (11.2%) | 19 (16.5%) | 0.407 |
| Artificial liver treatment | 23 (11.8%) | 5 (6.2%) | 18 (15.7%) | 0.045 |
| HR (bpm) | 82.66± 31.17 | 85.60 ± 45.47 | 80.62 ± 14.50 | 0.273 |
| MAP | 90.51± 13.29 | 91.36 ± 13.33 | 89.91 ± 13.27 | 0.455 |
| WBC (×10^9^/L) | 4.30 (2.80, 5.70) | 4.10 (2.70, 5.32) | 4.40 (2.90, 5.85) | 0.499 |
| NLR | 3.00 (1.93, 4.95) | 3.00 (1.87, 4.84) | 2.80 (2.00, 5.00) | 0.895 |
| Hb (g/L) | 117.25 ± 30.24 | 120.21 ± 29.01 | 115.18 ± 31.03 | 0.254 |
| PLT (×10^9^/L) | 75.00 (52.50, 123.00) | 79.00 (53.00, 133.50) | 72.00 (50.00, 113.50) | 0.556 |
| ALB (g/L) | 35.81 ± 7.15 | 35.57 ± 6.82 | 35.97 ± 7.40 | 0.699 |
| TBil (μmol/L) | 39.10 (19.50, 115.85) | 32.50 (18.50, 84.30) | 44.30 (19.55, 138.85) | 0.176 |
| Na+ (mmol/L) | 139.24 ± 3.96 | 139.65 ± 3.97 | 138.96 ± 3.94 | 0.232 |
| Cr (μmol/L) | 65.20 (55.15, 77.40) | 64.30 (56.35, 78.78) | 65.20 (54.95, 76.60) | 0.642 |
| PT (s) | 17.64± 10.89 | 16.96 ± 11.73 | 18.12 ± 10.29 | 0.464 |
| INR | 1.26 (1.12, 1.63) | 1.25 (1.13, 1.44) | 1.28 (1.12, 1.72) | 0.431 |
| MELD > 20 | 43 (22.1%) | 12 (15.0%) | 31 (27.0%) | 0.048 |
| **[Intraoperative](javascript:;) indicators** |  |  |  |  |
| Anhepatic phase (min) | 55.0 (46.00, 66.00) | 52.00 (46.00, 62.25) | 56.00 (47.50, 66.00) | 0.125 |
| Blood loss (L) | 1.00 (0.60, 2.00) | 1.00 (0.60, 1.55) | 1.20 (0.80, 2.00) | 0.084 |
| Plasma transfusion volume (L) | 0.96 (0.60, 1.44) | 0.94 (0.58, 1.38) | 1.06 (0.62, 1.56) | 0.213 |
| **Postoperative (POD 3) indicators** |  |  |  |  |
| HR (bpm) | 91.15 ± 16.85 | 86.24 ± 15.34 | 94.57 ± 17.07 | 0.001 |
| SBP (mmHg) | 127.95 ± 22.23 | 128.19 ± 23.40 | 127.79 ± 21.49 | 0.903 |
| SI | 0.73 ± 0.17 | 0.69 ± 0.16 | 0.76 ± 0.17 | 0.007 |
| Hyperensort | 27 (13.9%) | 7 (8.8%) | 20 (17.4%) | 0.086 |
| MAP | 90.04 ± 13.99 | 89.95 ± 14.65 | 90.11 ± 13.59 | 0.936 |
| WBC (×10^9^/L) | 10.63 ± 5.75 | 9.72 ± 4.68 | 11.26 ± 6.33 | 0.067 |
| NLR | 29.58 (17.33, 42.63) | 31.00 (17.06, 39.50) | 28.81 (17.83, 46.00) | 0.536 |
| Hb (g/L) | 92.41 ± 14.84 | 93.67 ± 14.66 | 91.53 ± 14.96 | 0.322 |
| PLT (×10^9^/L) | 69.96 ± 34.20 | 73.49 ± 37.15 | 67.51 ± 31.92 | 0.231 |
| ALB (g/L) | 36.61 ± 5.65 | 37.58 ± 5.32 | 35.94 ± 5.79 | 0.046 |
| TBil (μmol/L) | 78.60 (52.75, 124.80) | 63.70 (43.35, 101.00) | 91.00 (55.95, 143.05) | 0.007 |
| Cr (μmol/L) | 81.30 (66.85, 103.00) | 73.40 (64.58, 98.05) | 93.20 (72.25, 109.35) | 0.003 |
| INR | 1.45 (1.27, 1.67) | 1.33 (1.21, 1.51) | 1.57 (1.33, 1.75) | <0.001 |
| PaO_2_/FiO_2_<400 mmHg | 114 (58.5%) | 34 (42.5%) | 80 (69.6%) | <0.001 |
| Lac (mmol/L) | 2.90 (2.00, 4.90) | 2.50 (1.78, 3.85) | 3.60 (2.30, 5.95) | <0.001 |
| Glu (mmol/L) | 14.5 ± 4.80 | 13.47 ± 4.59 | 15.23 ± 4.83 | 0.012 |
| CRP (mg/L) | 84.70 (62.75, 112.40) | 81.35 (50.40, 108.40) | 86.80 (69.65, 114.50) | 0.134 |
| Blood FK506 concentration (ng/mL) | 6.93± 3.94 | 7.07 ± 4.11 | 6.33 ± 3.81 | 0.195 |
| Abnormal liver blood supply | 23 (11.8%) | 9 (11.3%) | 14 (12.2%) | 0.844 |
| Hydropericardium | 18 (9.2%) | 4 (5.0%) | 14 (12.2%) | 0.089 |
| Length of ICU stay (days) | 4.25 ± 4.28 | 3.65 ± 1.54 | 4.66 ± 5.39 | 0.105 |

HR, heart rate; MAP, mean arterial pressure; WBC, white blood count; NLR, Neutrophil to Lymphocyte ratio; Hb, hemoglobin; PLT, platelet; ALB, albumin; TBil, total bilirubin; PT, prothrombin time; INR, international normalized ratio; Cr, Creatinine; MELD, Model For End-stage Liver Disease; Lac, [lactic](javascript:;) acid; Glu, blood glucose; CRP, C-reactive Protein; PaO_2_, arterial partial oxygen pressure; FiO_2_, fraction of inspired O_2_; PaO_2_/FiO_2_, PaO_2_-to-FiO_2_ ratio; SBP, systolic blood pressure; SI, shock index= HR-to-SBP ratio; ICU, intensive care unit; FK506, tacrolimus.

Values are expressed as count (percentage), mean standard ± deviation or median (interquartile range).

Statistically significant P values are written in bold.
